# Beyond Rural Versus Urban: Referral Pathways and Structural Heart Programs May Influence Outcomes After Left Atrial Appendage Occlusion

**DOI:** 10.1002/clc.70412

**Published:** 2026-07-07

**Authors:** Teruhiko Imamura

**Affiliations:** ^1^ Second Department of Internal Medicine University of Toyama Toyama Japan

**Keywords:** referral pathway, rural health, structural heart disease

To Editor,

I read with great interest the article, evaluating inpatient outcomes following left atrial appendage occlusion (LAAO) in rural versus urban hospitals across the United States [1]. The adjusted inpatient mortality and complication rates following LAAO were comparable despite substantial differences in procedural volume. Several concerns have been raised.

Referral pathways may substantially influence the observed comparison between rural versus urban hospitals. Patients undergoing LAAO at rural hospitals may represent a carefully selected population with relatively straightforward anatomy and lower anticipated procedural complexity, whereas patients with challenging left atrial appendage morphology, multiple comorbidities, or greater procedural risk may be preferentially referred to tertiary urban centers [2]. Consequently, hospital location may partly represent differences in referral patterns rather than intrinsic differences in procedural performance. This possibility may also explain why unadjusted complication rates were numerically higher in urban hospitals despite their greater procedural experience. Future studies evaluating referral networks, interhospital transfer patterns, or characteristics of patients referred from rural to urban institutions may provide valuable insight into this issue.

The higher frequency of discharge to acute care facilities among rural patients deserves additional discussion [1]. Although inpatient mortality and procedural complications were comparable after adjustment [1], the greater need for transfer following discharge may represent differences in post‐procedural care pathways rather than differences in procedural safety. Rural hospitals may appropriately perform LAAO while relying on affiliated facilities for continued monitoring, rehabilitation, or management of complex comorbid conditions [3]. Accordingly, discharge disposition may reflect regional organization of healthcare delivery and resource allocation rather than adverse procedural outcomes alone. Understanding these post‐procedural care pathways may be pivotal when evaluating equity in structural heart disease care.

The comparison based solely on rural versus urban hospital location may not fully capture institutional expertise in contemporary LAAO practice. LAAO has increasingly become integrated into comprehensive structural heart disease programs alongside trans‐catheter aortic valve replacement, trans‐catheter mitral interventions, and other advanced catheter‐based interventions. The availability of multidisciplinary heart‐valve teams, dedicated cardiac imaging specialists, standardized patient selection protocols, and experienced peri‐procedural support may have a greater influence on clinical outcomes than geographic location itself [4]. Future investigations incorporating measures of structural heart program maturity or multidisciplinary infrastructure may help identify modifiable institutional factors associated with optimal outcomes.

## Funding

The author has nothing to report.

## Ethics Statements

The author has nothing to report.

## Consent

The author has nothing to report.

## Conflicts of Interest

The author declares no conflicts of interest.

## Data Availability

Data sharing is not applicable to this article as no datasets were generated or analyzed during the current study.
